# Sensitivity of the lateral flow urine lipoarabinomannan assay in ambulant adults with advanced HIV disease: data from the TB Fast Track study

**DOI:** 10.1093/trstmh/traa018

**Published:** 2020-04-20

**Authors:** Mpho Tlali, Katherine L Fielding, Aaron S Karat, Christopher J Hoffmann, Tshifhiwa Muravha, Alison D Grant, Salome Charalambous

**Affiliations:** The Aurum Institute, Johannesburg, South Africa; TB Centre, London School of Hygiene & Tropical Medicine, Keppel Street, London, WC1E 7HT, UK; School of Public Health, University of the Witwatersrand, Johannesburg, South Africa; TB Centre, London School of Hygiene & Tropical Medicine, Keppel Street, London, WC1E 7HT, UK; Johns Hopkins University School of Medicine, Baltimore, MD, USA; The Aurum Institute, Johannesburg, South Africa; TB Centre, London School of Hygiene & Tropical Medicine, Keppel Street, London, WC1E 7HT, UK; School of Public Health, University of the Witwatersrand, Johannesburg, South Africa; Africa Health Research Institute, KwaZulu-Natal, South Africa; School of Nursing and Public Health, University of KwaZulu-Natal, Durban, South Africa; The Aurum Institute, Johannesburg, South Africa; School of Public Health, University of the Witwatersrand, Johannesburg, South Africa

**Keywords:** algorithms, lipoarabinomannan, South Africa, TB, TB diagnostic tests, HIV

## Abstract

**Background:**

WHO guidelines recommend the lateral flow urine lipoarabinomannan assay (LF-LAM) for TB diagnosis in hospitalised HIV-positive individuals. The role of LF-LAM among ambulant patients remains less well defined. We investigated the sensitivity of LF-LAM among ambulant HIV-positive adults in primary health clinics in South Africa.

**Methods:**

We enrolled adults (aged ≥18 y) with CD4 counts of ≤150 cells/mm^3^ who had not received TB treatment or antiretroviral therapy in the preceding 3 or 6 mo, respectively. Research nurses performed the LF-LAM test on freshly voided urine. Results were compared with a reference standard of positive mycobacterial culture (sputum or urine).

**Results:**

Of 1505 (54.5% female; median age 37 y; median CD4 count 73 cells/mm^3^) participants, 973 (64.7%) had a mycobacterial culture result; 105/973 (10.8%) were positive for *Mycobacterium tuberculosis*. LF-LAM sensitivity was 41.9% (95% CI 32.3 to 51.9%) and 19.0% (95% CI 12.0 to 27.9%) using grade 1+ and grade 2+ cut-off points, respectively. Sensitivity increased with severe immunosuppression and in the presence of poor prognostic indicators (low haemoglobin, body mass index).

**Conclusions:**

When used as the only TB diagnostic test, LF-LAM sensitivity is suboptimal, particularly using the grade 2+ cut-off. More sensitive tests for TB are needed that can be used in primary care settings.

## Introduction

TB remains the leading cause of death among HIV-positive people in low- and middle-income counties (LMIC).^1^ TB is often missed and untreated in people with advanced HIV disease.^2^^,^^3^ The challenge of diagnosing TB in people with advanced HIV is multifactorial, including inherent delays of mycobacterial culture (the gold standard diagnostic test), reliance on suboptimal sputum-based testing, high risk of extrapulmonary TB and sputum scarcity. The development of the lateral flow urine lipoarabinomannan assay (LF-LAM) represented an opportunity to address some of these challenges: it is a point-of-care test that detects lipoarabinomannan (LAM), a lipopolysaccharide excreted in urine from an active or degenerating mycobacterial cell wall that is released in people with active TB disease. The assay is appropriate for use in LMIC because urine is an easy and accessible specimen to collect, with a lower infection risk than sputum. The results of the assay are rapid, read after 25 min by comparing the test strip with a manufacturer-provided reference card. Importantly, in January 2014, the manufacturer changed the reference card supplied with the assay from the original five intensity bands to four intensity bands, thereby dropping the grade 1+ cut-off point on the original reference card so that a positive result is now represented by the old grade 2+ cut-off only. The effect of this meant that a very low concentration of LAM on the test strip would be interpreted as a negative test result rather than a positive result. This change resulted in an increase in specificity at the cost of loss of sensitivity.

**Figure 1:**
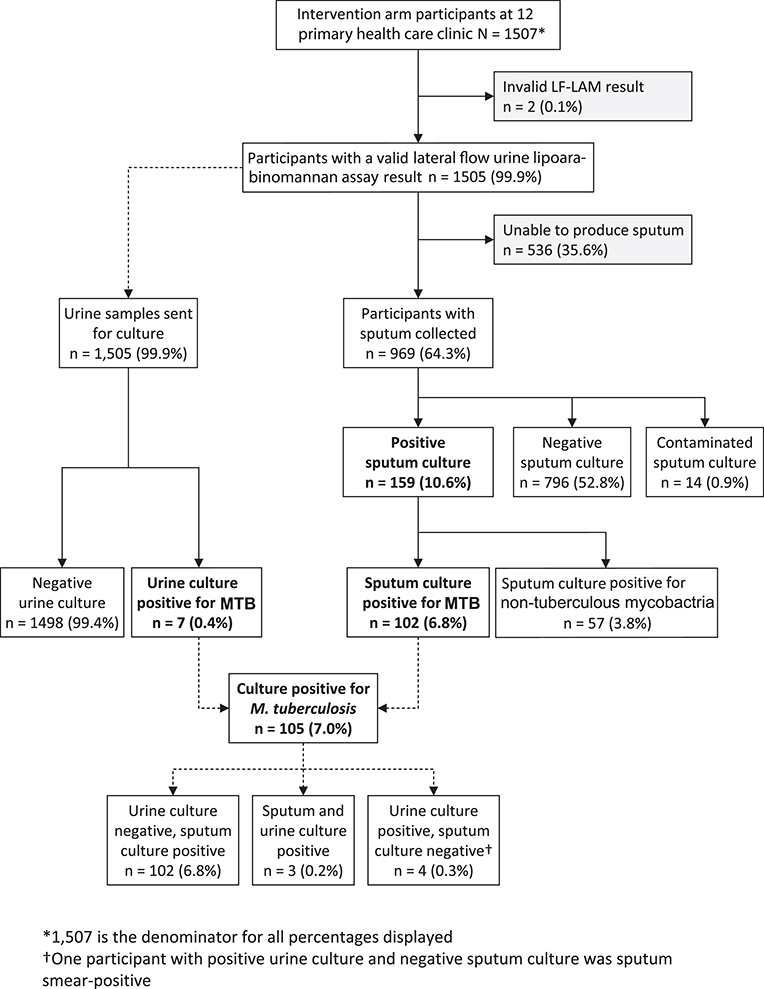
TB Fast Track intervention arm study participant profile. MTB, Mycobacterium tuberculosis.^*^1507 is the denominator for all percentages displayed. ^†^One participant with positive urine culture and negative sputum culture was sputum smear-positive.

In 2015, the WHO released guidelines^4^ for using the LF-LAM assay (informed in part by preliminary results from our study^5^), which recommended use of the assay only in HIV-positive adults, specifically among those who are hospitalized with TB symptoms and CD4 counts of <100 cells/mm^3^, or those who are ‘severely ill’. Evidence for the guidelines was derived from several studies that evaluated LF-LAM in hospitalized HIV-positive patients. Fewer studies evaluating the performance of LF-LAM have been conducted in ambulant patients, therefore the utility of the test in outpatient populations remains uncertain.

We report on a subanalysis of the TB Fast Track trial^6^ assessing the sensitivity of LF-LAM among ambulant, HIV-positive adults recruited to the intervention arm of the study.

## Methods

The TB Fast Track trial was a pragmatic, open, cluster-randomised trial conducted in 24 peri-urban and rural primary healthcare clinics in the Gauteng, North West and Limpopo provinces, South Africa, where HIV services were predominately nurse-led. Recruitment to the study took place between 19 December 2012 and 18 December 2014. The study design has previously been described.^7^ The intervention was nurse-initiated empirical TB treatment, guided by a point-of-care, technology-based algorithm, which included body mass index (BMI), haemoglobin and LF-LAM.

HIV-positive adults (aged ≥18 y) with a CD4 count of ≤150 cells/mm^3^ were included. The main exclusion criteria were: any TB treatment in the preceding 3 mo; antiretroviral therapy (ART) in the preceding 6 mo; or too sick to be managed in ambulant care. Too sick was defined as any of: temperature >39°C; respiratory rate >30 breaths/min; heart rate >120 beats/min; systolic blood pressure >180 or <90 mmHg; or any other medical condition necessitating immediate hospital referral.

At enrolment, research nurses collected data on demographics and medical history, including using the WHO TB screening tool to assess symptoms suggesting TB (cough, weight loss, night sweats or fever of any duration)^8^; measured height and weight; took a finger-prick blood sample for haemoglobin (Hemocue 201+, Hemocue, Angelholm, Sweden); and tested a freshly voided urine sample with LF-LAM, as per the manufacturer’s instructions. After 25 min, the LF-LAM result was read by comparing the test strip with the manufacturer's pre-January 2014 reference card, with five intensity-level bands. In this analysis, we show results both for grade 1+ cut-off, as recommended as of pre-January 2014, and for grade 2+ cut-off, which corresponds to the current recommended cut-off on the new reference cards.

A single, spontaneous, spot sputum was collected from participants who were able to provide a sputum sample and this was cultured for mycobacteria using the BACTEC MGIT 960 system (Becton Dickinson Microbiology Systems, Cockeysville, MD, USA). Positive cultures were assessed for species identification and drug susceptibility using the GenoType Hain MTBDRplus assay (Hain Lifescience GmbH, Germany). Urine samples collected at enrolment and frozen at −80°C were tested for mycobacterial culture at the end of the study to strengthen the gold standard, particularly for participants unable to produce sputum or with extrapulmonary disease.

The inclusion criteria for this analysis were all the intervention arm trial participants with valid LF-LAM (a visible band in the control window of the test strip) and sputum or urine culture results. Participants who were sputum or urine culture positive for non-tuberculous mycobacteria were considered negative in this analysis.

We estimated the sensitivity of LF-LAM against a reference standard of a positive culture for *Mycobacterium tuberculosis* (MTB) from either a single sputum or urine specimen. Data were summarised using frequencies for categorical data and medians and interquartile ranges (IQRs) for continuous data. The sensitivity of LF-LAM was summarised with exact binomial 95% confidence intervals (CIs). CI estimation using robust standard errors was also conducted, adjusting for clustering at the clinic level. LF-LAM sensitivity was summarised overall and stratified by haemoglobin, BMI and CD4 count. Sensitivities were compared across these strata using Fisher's exact test. Statistical analysis was conducted using Stata 13 (StataCorp LP, College Station, TX, USA).

## Results

Among 1507 intervention arm participants, 1505 (99.9%) had a valid LF-LAM result. The 1505 participants were predominantly female (820/1505 [54.5%]) and the median age was 37 (IQR 32–44) y. At least one WHO TB symptom was reported for 1095/1505 (72.8%); two or more symptoms were reported for 662/1505 (44.0%). The median CD4 count was 73 (IQR 35–111) cells/mm^3^; 508/1505 (33.8%) had a CD4 of ≤50 cells/mm^3^; 320/1505 (21.3%) had a BMI <18.5 kg/m^2^; and 428/1505 (28.4%) had a haemoglobin concentration of <10 g/dL.

Of the 1505 valid LF-LAM results, 1324 (88.0%) were negative (grade 0), 124 (8.2%) were grade 1, 20 (1.3%) were grade 2, 14 (0.9%) were grade 3, nine (0.6%) were grade 4 and 14 (0.9%) were grade 5. The numbers of participants with positive LF-LAM were 181/1505 (12.0%) and 57/1505 (3.8%) for grade 1+ and grade 2+ cut-off points, respectively.

### Sputum and urine culture results

A spot sputum specimen was collected in 969/1505 (64.4%) participants (Figure 1). Of these, 102 (10.5%) were culture positive for *M. tuberculosis*, 853 (88.0%) were culture negative for *M. tuberculosis* and 14 (1.4%) samples were contaminated. Seven intervention arm participants had a urine sample that was *M. tuberculosis* culture positive. Four had a bacteriologically positive sputum result at enrolment (three culture positive, one smear positive but culture negative); the remaining three were unable to provide sputum. The final yield of culture-positive *M. tuberculosis* from either sputum or urine or both was 105/973 (10.8%).

Among the 105 participants who were sputum culture positive for *M. tuberculosis*, LF-LAM was positive, using a grade 1+ cut-off, in 44/105 (sensitivity 41.9% [95% CI 32.3 to 51.9%]). Using a minimum grade 2+ cut-off, LF-LAM was positive in 20/105 (sensitivity 19.0% [95% CI 12 to 27.9%]; Table 1). Among 1389 participants who were culture negative or unable to produce sputum, 37 had a positive LF-LAM test. Results were similar when using robust standard errors to adjust for clustering at the clinic level.

**Table 1:** Determine TB LAM sensitivity among sputum and urine culture positive patients for *Mycobacterium tuberculosis* (MTB) (n=105) overall and stratified by body mass index (BMI), haemoglobin (Hb) and CD4 count

	LF-LAM positive (≥2+)			LF-LAM positive (≥1+)		
	n/N	% (95% CI)	p-value^*^	n/N	% (95% CI)	p-value^*^
Overall	20/105	19.0 (12.0 to 27.9)		44/105	41.9 (32.3 to 51.9)	
Hb ≤10 g/dL	14/47	29.8 (17.3 to 44.9)	0.01	25/47	53.2 (38.0 to 67.9)	0.05
Hb >10 g/dL	6/58	10.3 (3.9 to 21.1)		19/58	32.8 (21.0 to 46.3)	
BMI <18.5 kg/m^2^	9/38	23.7 (11.4 to 40.2)	0.44	20/38	52.6 (35.8 to 69.0)	0.10
BMI ≥18.5 kg/m^2^	11/67	16.4 (8.5 to 27.5)		24/67	35.8 (24.5 to 48.5)	
CD4 ≤50 cells/mm^3^	10/37	27.0 (13.8 to 44.1)	0.19	18/37	48.6 (31.9 to 65.6)	0.10
CD4 51–100 cells/mm^3^	7/37	18.9 (8.0 to 35.2)		18/37	48.6 (31.9 to 65.6)	
CD4 101–150 cells/mm^3^	3/31	9.7 (2.0 to 25.8)		8/31	25.8 (11.9 to 44.6)	

LF-LAM, lateral flow urine lipoarabinomannan.

^*^Fisher's exact test, comparing sensitivities by Hb, BMI and CD4 strata.

We further stratified LF-LAM sensitivity by subgroups at highest risk for mortality, those with BMI <18.5 kg/m^2^, haemoglobin <10 g/dL or CD4 <50 cells/mm^3^ (Table 1). Using a positive LF-LAM defined by the grade 1+ cut-off, the highest sensitivity was found in those with haemoglobin <10 vs ≥10 g/dL (53.2 vs 32.8%; p=0.05), BMI <18.5 vs ≥18.5 kg/m^2^ (52.6 vs 35.8%; p=0.10) and CD4 ≤50 vs 51–100 vs 101–150 cells/mm^3^ (48.6 vs 48.6 vs 25.8%; p=0.10).

## Discussion

The sensitivity of LF-LAM was poor in our study population of ambulant adults with advanced HIV disease who were not yet initiated on ART. Even after restricting the analysis to participants who were severely immunocompromised (CD4 <50 cells/mm^3^), LF-LAM sensitivity remained low. Overall sensitivity was reduced by almost half when the cut-off point was changed from grade 1+ to grade 2+. This is a substantial reduction for an already suboptimal result.

The role of the LF-LAM test among hospitalised HIV-positive patients has been well established but the role of the test among ambulant patients remains less well defined. Our results are among few studies reporting the performance of LF-LAM in ambulant HIV-positive patients. Other outpatient evaluations of the LF-LAM test report a wide range in sensitivities between 12.5 to 41% (grade 1+)^9^^,^^10^ and 5.4 to 58% (grade 2+).^9^^,^^11–13^ This difference reflects the differences in the participant selection, the reference standards used and whether testing was done to screen for or diagnose TB.

In the recent meta-analysis by Bjerrum et al., pooled sensitivity estimates for the LF-LAM test among outpatients ranged from 17.0 to 47.0% at grade 2+ cut-off.^13^ The four outpatient studies included in this estimate used culture-positive *M. tuberculosis* (MTB) or Xpert MTB/RIF as the reference standard for diagnosis of TB. Our grade 2+ cut-off sensitivity of 19.6% falls at the lower end of this range; however, two studies in the meta-analysis excluded participants without signs or symptoms of TB and one study included severely ill patients.

Among our study participants, 73.0% reported >1 TB symptom and 44.0% ≥2 TB symptoms. A much higher sensitivity of 58.0% at the grade 2+ cut-off was reported by Huerge et al.^14^ using a positive Xpert MTB/RIF and MTB culture for the reference standard. Only participants reporting ≥2 wk of TB symptoms were included in the study. The median CD4 count was 88 cells/mm^3^ and almost 60% of participants met the study criteria for ‘severely ill’ (criteria similar to those used in our study to exclude participants). The lowest sensitivities are reported by Hanifa et al.,^9^ 5.4% at grade 2+ cut-off among participants with confirmed TB (sputum culture or Xpert MTB/RIF positive). This is despite a study population with a low median CD4 count (111 cells/mm^3^) and frequently reported TB symptoms (53%). The important difference in this study is that the test was used as a screening tool for participants already established in care, with more than half (53%) taking ART.

Our study contributes valuable data from a large, well characterised cohort of ambulant adults with advanced HIV disease and poor prognostic indicators who would be an ideal population for a highly sensitive non-sputum-based TB point-of care test. Additionally, in our study, LF-LAM was performed by trained research nurses, working under normal clinic conditions that closely approximated the real-life setting in which the test would be used.

Our analysis was limited because of the suboptimal reference standard used. Only a single spot sputum and urine culture specimen were collected to facilitate the pragmatic design of the parent study. As a result, we may have overestimated the sensitivity of the LF-LAM test. We do not report on the specificity of the test, as the pragmatic study design involved minimal TB diagnostic testing and did not allow us to determine with certainty which participants did not have active TB at enrolment.

### Conclusion

Based on findings from this study, LF-LAM is not sensitive enough to be used as the only test for TB diagnosis in an outpatient population, even among a very sick ambulant population.

The current recommended 2+ cut-off reduced the sensitivity of LF-LAM substantially. Newer formulations of the LF-LAM test that incorporate an additional concentration step to increase sensitivity may have better potential, particularly for people unable to produce sputum.
